# Preferred Characteristics for mHealth Interventions Among Young Sexual Minoritized Men to Support HIV Testing and PrEP Decision-Making: Focus Group Study

**DOI:** 10.2196/51103

**Published:** 2023-10-16

**Authors:** Juan Pablo Zapata, Sabina Hirshfield, Kimberly Nelson, Keith Horvath, Steven A John

**Affiliations:** 1 Institute for Sexual and Gender Minority Health and Wellbeing Northwestern University Chicago, IL United States; 2 State University of New York Downstate Health Sciences University Brooklyn, NY United States; 3 Department of Medicine School of Public Health Boston University Boston, MA United States; 4 Department of Psychology San Diego State University San Diego, CA United States; 5 Department of Psychiatry and Behavioral Medicine Medical College of Wisconsin Milwaukee, WI United States

**Keywords:** mobile health, eHealth, minority health, male adolescents, HIV prevention, sexual health, support, HIV testing, prevention, decision-making, men, sexual minority men, youth, adolescent, mobile simulation, virtual simulation, user-friendly, design, implementation, mobile phone

## Abstract

**Background:**

Epidemiological trends in the United States have shown an increase in HIV cases among young sexual minoritized men. Using mobile health (mHealth), which refers to health services and information delivered or enhanced through the internet and related technologies, is a crucial strategy to address HIV disparities. However, despite its potential, the practical implementation of mHealth remains limited. Additionally, it is important to consider that young individuals may become accustomed to, distracted from, or lose interest in these apps, highlighting the need for regular updates and monitoring of relevant content.

**Objective:**

In this study, we sought to highlight the voices of young sexual minoritized men aged 17-24 years and explored preferred mHealth intervention characteristics and willingness to adopt these technologies among a diverse, nationwide sample of young sexual minoritized men.

**Methods:**

From April to September 2020, we recruited participants through web-based platforms such as social media and geosocial networking apps for men. These individuals were invited to participate in synchronous web-based focus group discussions centered around topics pertaining to HIV testing and prevention and their preferences for mHealth technologies.

**Results:**

A total of 41 young sexual minoritized men, aged between 17 and 24 years, participated in 9 focus group discussions spanning April to September 2020, with 3-7 participants in each group. The findings shed light on three key insights regarding young sexual minoritized men’s preferences: (1) the need for personalized and representative content, (2) a preference for mobile and web-based simulation of prevention scenarios, and (3) a preference for digital software with individually tailored content. As expected, preference for mHealth apps was high, which supports the potential and need to develop or adapt interventions that use smartphones as a platform for engaging young sexual minoritized men in HIV prevention. This study expands on previous research in multiple meaningful ways, delving into the use and perceptions of mHealth information amid the COVID-19 pandemic. This study also highlighted the importance of streamlined access to health care providers, especially in light of the barriers faced by young people during the COVID-19 pandemic. In terms of presentation and navigation, participants favored a user-friendly design that was easy to use and appropriate for their age, which was effectively addressed through the implementation of web-based simulations.

**Conclusions:**

Ultimately, this study provides valuable insight into the preferences of young sexual minoritized men when it comes to mHealth interventions and highlights the need for further research in order to develop effective and tailored HIV prevention tools. A future direction for researchers is to evaluate how best to address participants’ desire for personalized content within mHealth apps. Additionally, as technology rapidly evolves, there is a need to re-assess the effectiveness of web-based simulations, particularly those that are used in HIV prevention.

## Introduction

Ending the HIV epidemic in the United States is a federal priority [1] and social justice issue due to the disproportionate burden of HIV among marginalized populations [2]. Despite decreasing annual rates of new HIV diagnoses overall, among sexual minoritized men—including gay, bisexual, queer, and other men who have sex with men—new HIV diagnoses remain high, with approximately 25,000 diagnoses annually, comprising 70% of new HIV cases overall [3]. In particular, young sexual minoritized men aged 13-24 years make up 21% of all new HIV diagnoses; however, nearly half of all youths living with HIV are not aware they are living with HIV [2]. Improved implementation of HIV testing and prevention methods are urgently needed to combat the ongoing HIV epidemic in the United States [1].

HIV self-testing (HIVST) [4] is one mechanism to engage young sexual minoritized men early in the prevention cascade. Benefits of HIVST include convenience, confidentiality, privacy, and avoidance of HIV clinic stigma [5-15]. The use of HIVST became especially evident during the COVID-19 pandemic, when access to clinic-based HIV testing was severely limited and many young sexual minoritized men engaged in HIVST for the first time [16]. Nonetheless, financial and logistical barriers to HIVST are significant, as the most purchased over-the-counter option, OraQuick (OraSure Technologies), has a retail cost of US $20-$48 and is limited to those who are aged 17 years or older [4].

Upon completion of HIV testing, young sexual minoritized men are at a critical junction of entrance into the HIV continuum of care or continued engagement in HIV prevention. Linking individuals who receive a preliminary reactive HIV test result to confirmatory testing and care can improve clinical outcomes and reduce onward HIV transmission [17], yet standard packaging and instructions for HIVST are limited; thus, additional pretesting guidance is needed to encourage successful HIV testing and linkage to care [18]. Some young sexual minoritized men who test HIV-negative with HIVST would benefit from HIV pre-exposure prophylaxis (PrEP) [19-21]. Since receiving Food and Drug Administration approval in 2012, the modes of PrEP for HIV prevention include dosing options such as once-daily oral pills, on-demand oral pills, or a long-acting injectable every 8 weeks [22]. Nonetheless, PrEP uptake among young sexual minoritized men has been suboptimal, with fewer than 17% of young sexual minoritized men reporting PrEP use in 2019 [23] despite research indicating nearly 44% of young sexual minoritized men could benefit from PrEP based on federal guidelines [21-23].

Leveraging the internet to support implementation strategies focused on expanding HIVST and PrEP use is an impactful mechanism with large potential reach, especially since young sexual minoritized men already report seeking sexual health information on the internet [24-26]. Prior research has shown that many in-person interventions can be adapted using mobile health (mHealth) technology for health behavior change [27-32]. While in-person intervention strategies have several benefits (eg, building rapport), mobile-based technologies, such as apps and text messages, can provide access to individuals not linked to prevention and treatment care [33,34] and offers several key advantages, including confidentiality*,* intervention scalability*,* and delivery efficiency [35]. However, systematic reviews of mHealth apps for HIV prevention and treatment highlight that most of these apps were considered of low quality by users, as they did not have desirable features [33]. Further, studies on the general adoption and sustainability of mHealth apps show that despite a vast range of apps, only a small number are actually used and implemented [34].

Understanding the best ways to leverage mHealth to increase uptake and adherence to biomedical technologies for HIV prevention, including HIVST and PrEP, is critical to the development of feasible, acceptable, and effective mHealth approaches. Prior research has demonstrated several design and content-related considerations. Using qualitative interviews and focus groups, studies have found that sexual minoritized men perceive the focus on men who have sex with men behavior or on gay men deter engagement among some sexual minoritized men who are not open about their sexual orientation, and thus do not want to be seen on an app that is promoted for gay men [35,36]. Further research indicates that concerns regarding privacy when it comes to collecting sensitive information, such as behaviors related to sexual activity and drug use, can hinder the willingness of sexual minoritized men to use and engage with HIV prevention mHealth apps [34]. Additionally, it is important to consider that young people may become habituated to, distracted from, or bored with these apps, highlighting the need for regular updates and monitoring of relevant content [37].

We report findings from national web-based focus group discussions conducted with young sexual minoritized men in the United States on HIV prevention topics. The overarching goal of this investigation was to assess attitudes, beliefs, and willingness to use HIV mHealth technologies for young sexual minoritized men and to inform the design and development of an mHealth app aimed at improving engagement with HIVST, PrEP, and other HIV biomedical prevention technologies.

## Methods

### Participants and Procedures

As described previously [16,21,38], participants were recruited on the internet from social media and men-for-men geosocial networking apps between March and September 2020 to participate in web-based synchronous focus group discussions centered around topics of HIV testing and prevention. All ads featured images of 2 men, including a variety of young adult couples of various races and ethnicities. Fraudulent responses to the screening survey were minimized by excluding any information on eligibility criteria from study advertisements and referral mechanisms, using the “prevent ballot box stuffing” feature in Qualtrics (Qualtrics) to prevent multiple responses, offering no incentive for completion of the brief (5-10 min) screening survey and its associated web-based consent procedure, and using a delayed invitation procedure for the internet focus group discussions to avoid attempts at determining this study’s eligibility criteria [39]. To further ensure data integrity, duplicates were checked using a procedure of comparing contact information (ie, name, email, and phone number) and IP addresses.

To be eligible to participate in a web-based focus group discussion, participants were required to (1) be aged 17-24 years; (2) identify as male (inclusive of transgender men); (3) report one or more male sexual partners in the past 6 months, including those who identified as transgender; (4) self-report HIV-negative or unknown status; (5) report sexual behavior meeting US Centers for Disease Control and Prevention guideline criteria for PrEP [40], which include past-6-month behaviors including condomless anal sex (CAS) with a casual male partner, CAS with a main partner living with HIV or of unknown status, CAS with an HIV-negative main partner who reports CAS with other male partners, or having a recent history of bacterial sexually transmitted infection; and (6) reside in the United States. Individuals who screened as eligible received an email invitation to participate. Eligible participants who replied to our email invitation were then asked to complete a web-based consent procedure. Agreement to participate was obtained through Qualtrics using a guided procedure that described this study’s purpose, procedures, and other critical components. Participants were encouraged to email or call to obtain clarification on any questions prior to continuing, and several participants emailed with questions about privacy protections. Participants then completed a brief quiz to ensure adequate comprehension of the critical components of consent, including the voluntary nature of this study, risks and benefits to participation, and confidentiality of all data collected. Participants then agreed to participate on the internet, and a copy of this study’s informational letter was emailed to the address of their choosing. A waiver of guardian assent was obtained for those considered minors. Participants were then scheduled for web-based group discussions, with 6-12 individuals invited per group.

In total, 9 synchronous web-based focus groups were administered by a team of 2-3 researchers via web-based chat. Specifically, we used the real time web-based meeting platform Adobe Connect (Adobe Systems) for the web-based group discussions, which allowed between-participant anonymity with preselected usernames and no video recording. Web-based focus group discussions are a valid method of qualitative data collection for sensitive topics, which maintain fidelity of themes identified compared to in-person focus group discussions [41,42]. Web-based focus groups were about 90 minutes in duration, and participants were compensated with a US $40 e-gift card. All text data were saved for analysis.

### Ethics Approval

This study’s protocol was approved by the Medical College of Wisconsin (PRO00034897). Before beginning this study, young sexual minoritized men provided informed consent, which was appropriately documented. Throughout this study, participants’ confidentiality was carefully maintained at all times.

All procedures performed in studies involving human participants were in accordance with the ethical standards of the institutional or national research committee and with the 1964 Helsinki declaration and its later amendments or comparable ethical standards.

This study met the Medical College of Wisconsin institutional review board’s definition of “minimal risk” and a waiver of informed consent was granted. All participants agreed to participate after completion of a guided procedure using Qualtrics that described this study’s purpose, procedures, and other critical components, as well as a capacity-to-consent procedure. A waiver of guardian permission was obtained for those considered minors.

### Focus Group Content

A semistructured focus group guide was developed to elicit preferences, opinions, barriers, facilitators, and decision-making about a variety of topics related to HIV testing and prevention, including HIV testing and self-testing, PrEP, postexposure prophylaxis, mHealth intervention characteristics, and web-based focus group discussion experiences. Data used for this analysis centered around data on preferred mHealth intervention characteristics. More specifically, participants were provided with the following introduction and prompts:

We are working on developing an online program to help people like you make informed decisions about HIV testing and methods of HIV prevention including PrEP and PEP [post-exposure prophylaxis]. We’d like your input to help us better design this program. What could we do to help make participation in an online, HIV prevention study appealing to people like you? What types of material would you or others like to see about PrEP and PEP in an online study? How do you think people will want this content delivered online?

### Data Analysis

Descriptive statistics were used to characterize the sample using screening survey data. Transcripts were coded using MAXQDA (VERBI GmbH) by authors JPZ and SAJ, who are trained in qualitative methods using a 3-stage analytic coding strategy including open, axial, and selective coding [43]. First, a list of a priori codes was developed in advance by the first and second authors on topics addressed by the focus group guide. Codes were created by noting overlapping concepts in the transcripts and developing code definitions that represented the data. Each transcript was coded and reviewed separately to ensure adequate application of codes. During the initial analytic phase, each analyst separately coded the same randomized transcript with the codebook and inconsistencies were discussed until an agreement was reached. Coded focus groups were then analyzed using thematic content analysis [44] to highlight patterns and identify the meaning of the data.

## Results

### Overview

Table 1 shows the demographic characteristics of the participants. A total of 41 young sexual minoritized men aged between 17 and 24 years participated in 9 focus group discussions spanning April to September 2020, with 3-7 participants in each group. Focus group participants were predominantly (n=35; 85%) cisgender men and self-identified as gay (n=27, 66%) or bisexual (n=12, 29%). The sample was 27% (n=11) Black and 29% (n=12) Latino, and about half (n=22, 54%) reported their relationship status as single. Among the participants, 32 were recruited from social media (78%) and 9 (22%) were recruited from geosocial networking apps. All the participants owned smartphones and had access to a personal computer.

**Figure 1:**
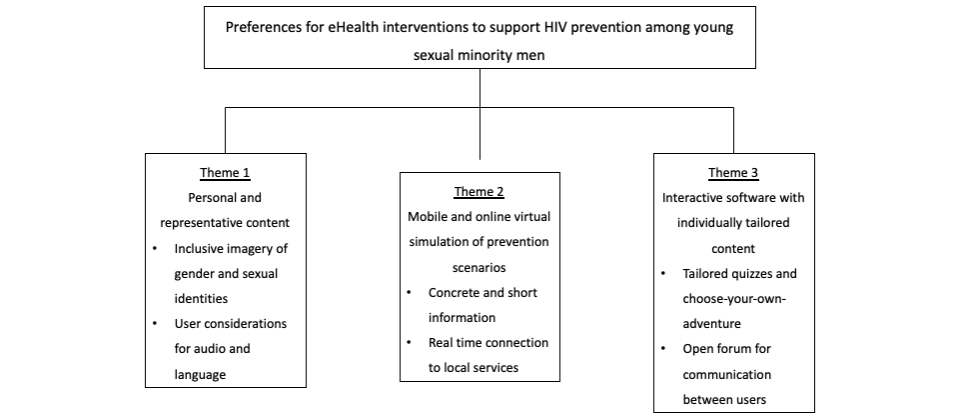
Themes and subcategories.

The findings provided insight on (1) young sexual minoritized men’s need for personal and representative content, (2) their preference for mobile and web-based simulation of prevention scenarios, and (3) their preference for digital software with individually tailored content. Main categories and subcategories are illustrated in Figure 1.

**Table 1:** Demographics characteristics of young sexual minoritized men (N=41). Percentages may not add up to 100 due to rounding.

Characteristics				Values
**Continuous variable** **, mean (SD)**				
Age (years; range 17-24 years)				21.0 (2.5)
**Categorical variables, n (%)**				
	**Gender identity**			
		Cisgender man	35 (85)	
		Transgender man	6 (15)	
	**Race or ethnicity**			
		Black, non-Hispanic	11 (27)	
		Latino or Hispanic	12 (29)	
		White, non-Hispanic	14 (34)	
		Multiracial or another	4 (10)	
	**Sexual orientation**			
		Gay	27 (66)	
		Bisexual	12 (29)	
		Queer	2 (5)	
	**Relationship status**			
		Single	22 (54)	
		Partnered	19 (46)	
	**PrEP^a^ use status**			
		Never	25 (61)	
		Prior PrEP use	7 (17)	
		Current PrEP use	9 (22)	
	**HIVST^b^ use status**			
		Never	11 (27)	
		Prior HIVST use	30 (73)	
	**Region**			
		Midwest	12 (29)	
		Northeast	11 (27)	
		South	12 (29)	
		West	6 (15)	
	**Recruitment source**			
		Social media	32 (78)	
		Men-for-men geosocial networking apps	9 (22)	

^a^PrEP: pre-exposure prophylaxis.

^b^HIVST: HIV self-testing.

### Theme 1: Personal and Representative Content

Nearly all participants when asked about their input on how to design a web-based program for HIV prevention reported a need for personalized content. Those who reported past use of mHealth tools such as personal computers or mobile phones to seek sexual health information expressed that many of these apps had unrealistic representation of young sexual minoritized men. For instance, a few participants found that sexual health education tended to be paired with muscular, thin, and light-skin-toned men. Many stated that they did not relate to the content and did not feel represented in the narratives presented. For instance, 2 participants stated the following:

LGBT and HIV websites usually have darkly lit photos of ripped guys with their shirts off. It is such a cliché. I bring this up because that’s what I see in New York.

It depends on the individual. I just think it’s important that the videos have real people who look like us and do not waste time with information that we can’t relate to.

Participants who had tried any health-related web-based program (eg, prior research, health apps, or similar) reported not finding any apps relevant enough for their immediate needs or backgrounds. These comments were of particular concern for some racial and ethnic minority participants who did not feel represented in LGBT or HIV web-based programs. Recommendations to design a web-based program were to have presenters with more diverse backgrounds. For example, 1 participant noted the following benefits:

I think representation of a wide array of racial identities in the videos would be helpful. It would make it easier to relate to the presenters and likely make learners more receptive to the information.

Additional suggestions were to have the materials in Spanish and easy enough to understand for people who do not have a medical background. In total, 1 participant further expressed their need for information to be accessible to youth with visual impairment. Similar concerns centered on user interface components, such that development should ensure that visual material (ie, graphs, charts, and diagrams) be programmed to let a screen reader read out image-based information or allow users to make changes to the font size and color.

Nearly all participants were positive toward or expressed their interest in sexual health information with digital technologies (eg, web-based multimedia, telephone, web-based reality, and social media). Nonetheless, many participants expressed their preferences for how the information is presented and by whom. Although some participants preferred to have a medical professional in the intervention, many were more interested in characters or presenters from their own background.

Having heavy presence with existing queer culture would be really important. Something they were doing pre-COVID was showing to different places with their app or video game, and they’d get people like Shangela or Valentina [famous drag queen entertainers] to make content for them which is how I first even heard about PrEP.

In the couple of online programs about HIV that I’ve been a part of, the graphics are really bad/outdated with random people. It was hard to focus on the information because it felt like it was designed by an 8th grader. I think it is important to have personalized information for HIV resources and organizations from familiar faces.

### Theme 2: Preference for Mobile and Web-Based Simulation of Prevention Scenarios

When asked about their preference for a web-based, HIV prevention program, young sexual minoritized men discussed their preference for web-based simulations of prevention scenarios. Many participants described their need for mHealth as a medium to improve access and awareness to HIV prevention techniques. Additionally, almost half of the participants mentioned a need for mobile and web-based simulations to support their communication with health care providers. Further, 1 participant discussed how these web-based simulations could facilitate conversations about which form of PrEP to consider with their provider:

Clinical videos that I can watch on my phone, having good concise videos available with conversations. To determine which type of PrEP would be best and knowing how to ask for it.

Themes related to web-based components fell into two categories: (1) web-based simulation with *concrete* and *short* information and (2) additional considerations for more *digital* prevention scenarios (theme 3). When reviewing the data, it became apparent that concerns and considerations differed among participants based on their preference for concise and immediate information, as opposed to a more digital medium. Conversely, some participants expressed a desire for additional components that would enhance their engagement with the app or website. Among participants, web-based simulation with concise and practical prevention scenarios emerged as the preferred choice across some focus group participants. This preference was driven by the desire for immediate access to local resources and information. Generally, these participants had limited time and motivation to engage with digital programming. Given the seriousness of the topic of HIV prevention, they gravitated toward more serious content. Further, 2 participants noted the following:

Interactive things could be more effective for younger guys, but I need something that feels more real and healthcare oriented. Something quick and online.

I am not against animation or something interactive, but I would be concerned that people would see it being too much of a video game and not as serious of a topic.

Additionally, participants expressed the importance of web-based simulations providing instant access to local prevention resources. They also desired integration of other relevant health information, such as consolidating COVID-19 testing details and HIV testing options. This aspect generated great enthusiasm among focus group participants, particularly those with limited access to reliable HIV resources. They discussed various barriers to testing, including transportation challenges and health literacy, and expressed a need for guidance on which clinics or health centers would be best suited for their specific concerns. By connecting users immediately and alleviating the burden of determining reliable information, this app would greatly benefit them.

Opinions specific to the length of the mHealth content were mixed among all participants. Most participants who preferred less compounded content, expressed their interest in shorter videos that were broken down by specific topics, citing their fatigue with web-based health information and communication. Participants recognized that with the widespread use of social media apps, such as with TikTok, many youths tended to give their attention to shorter videos. Some participants, further expressed how much of their attention span has changed since the COVID-19 pandemic and recommended shorter content to sustain their attention:

I have an extremely short attention span right now, so I may not survive 10 -15 minutes of information. Sometimes I stop YouTube videos that are 5 minutes because they lose my attention. 1-minute clips are great because if someone chooses to continue, they can but if they don’t, they haven’t missed anything, and are more intrigued to come back later.

I think people these days have short attention spans so maybe no more than what I would see on Tik-Tok.

### Theme 3: Preference for Digital Apps With Individually Tailored Content

When participants were asked for their opinion on avatar-based or choose-your-own-adventure apps, participants reported a wide range of different digital approaches. As mentioned in theme 2, participant responses were divided, with some favoring concise and immediate information, while others preferred digital apps with social features and personalized content. The most common digital apps discussed among participants were (1) individually tailored videos and quizzes, (2) choose-your-own-adventure design, and an (3) open communication forum. In the focus group discussions, participants noted that an app with individually tailored videos and quizzes would enable them to be more active in the process and conscious of their responses. Suggested features mainly had to do with how to ensure that the quizzes would produce relevant information and not overwhelm the user with pages of written text. For example, participants spoke passionately about their experience with different Buzzfeed quizzes and discussed how a similar format could increase their engagement with an app for sexual health. Further, 2 participants noted the following:

An interactive flowchart quiz so people could retake the quiz and see what different answers would result in.

Buzzfeed quiz format or a Tik-Tok based study that creates more personal material. Add an Olivia Rodrigo song and boom! Suddenly PrEP is fun.

As illustrated in theme 1, 1 principal feature in the design of an mHealth intervention is personalized content. This theme was further illustrated in discussions among participants for digital apps with a choose-your-own-adventure design. Participants further noted that in prior experiences with mHealth interventions, they were generally presented with “excessive medical information” that appeared to be “fear-mongering” or “shameful.” Instead, participants were very enthusiastic about a design that would allow them to explore their own sexual identity while learning more about sexual health. An idea was raised by participants to use an avatar that resembled the user, that would then be embedded within a web-based world. They suggested that the focus should not be entirely on HIV, but rather on relationships, sex in a positive matter, and subsequent content on HIV prevention. Further, 2 participants recommended the following:

Maybe, if you did like real life scenarios then talked about risks, different options to prevent risks with an Avatar you created. The videos would be helpful, though I know realistic conversations are hard to produce and often times come across as forced and cringy, so an Avatar like a Sims character could help.

Sexy* Adventure Scenarios are great. Queers don’t have enough of this fun content, and I would be more interested in something less forceful about HIV. it definitely would feel more personal.

The final component that emerged across focus groups were major considerations for a social forum that would allow the user to engage with other users. Although not specifically queried by the moderator, participants recommended an interface design similar to the one used in our study. They identified that using an open forum would make it easier to engage with the content in a more personalized and digital method. For many young sexual minoritized men, a platform such as the one used in our study, learning about HIV prevention methods happened as they were connecting with other study participants. As young sexual minoritized men learned about and connected with one another, they were exposed to accurate HIV information, testing stories, and services designed for young sexual minoritized men. Some participants also recommended a format similar to the one used on Reddit, which they cited as a source of information for PrEP. Finally, in their discussions of an open forum, participants generally liked that they could create a personalized name and recommended a similar design for an mHealth intervention. For example, 2 participants noted the following:

I would prefer to have an online chat or forum due to the confidentiality. If this were in a video format, then I wouldn’t have participated as much since I am not comfortable speaking about these topics with family around

Morgan, 23 years old

I would recommend an app or program designed like this. I learned something and I feel like I’m helping other people learn more about what young gay people think about and things that affect us most

Jake, 20 years old

## Discussion

### Principal Findings

Similar to past studies [25,30-35,45-47], in our study we conducted web-based focus groups with young sexual minoritized men to inform the subsequent development of an HIV-related mHealth app. However, this study expanded on the findings of prior studies in several significant ways. First, our focus groups explored the use of and attitudes toward mHealth information during the COVID-19 pandemic. Although the COVID-19 pandemic is no longer considered a public health emergency, some studies have illustrated the ways in which individuals’ attitude toward the use of web-based health information and communication technologies have changed [48]. Interestingly, there was a decline in downloads for health education apps during the COVID-19 pandemic among some populations [48]. Therefore, through formative work about the user’s needs and preferences is necessary to continue to guide the development of novel health-related apps and ensure that the content remains engaging and relevant to young sexual minoritized men.

This study complements the growing body of research to help us better understand how to make HIV-related mHealth interventions engaging and useful for young sexual minoritized men. Young sexual minoritized men continue to be disproportionately affected by HIV in the United States, despite various existing prevention efforts. While mHealth technologies represent powerful methods to recruit and deliver HIV prevention interventions and information, such as for HIVST and PrEP to young people [49], few studies have effectively developed or sustained these technologies to reach young sexual minoritized men [45]. Our study sought to understand the design features that could improve engagement among young sexual minoritized men with HIV-related mHealth apps and offer suggestions to researchers and practitioners who seek to use mobile and electronic technologies to engage this population.

As expected, preference for mHealth apps was high, which supports the potential and need to develop or adapt interventions that use smartphones as a platform for engaging young sexual minoritized men in HIV prevention. As with prior research, some young sexual minoritized men preferred simple, minimal, yet timely apps with immediate access to nearby resources for HIV testing and PrEP [45]. The finding of more streamlined access to health care providers was further supported by the barriers encountered by youths throughout the COVID-19 pandemic [16]. In terms of presentation and navigation, participants preferred a user-friendly design, specifically one that was easy to use and was age-appropriate and relevant, which was mainly addressed by the design of web-based simulations. Designers of mHealth apps should therefore consider a user-centered design [46], in which the implementation of new mHealth apps is driven by potential users. In addition, the design of an mHealth app should directly involve diverse young sexual minoritized men, and be inclusive of cultural, literacy, and linguistic needs. Inaccessible design features should also be considered for young sexual minoritized men with hearing and visual impairments, as there are currently no HIV prevention mHealth apps for young sexual minoritized men with disabilities. This demands close attention and action to ensure people with disabilities are not left behind [47].

Providing a secure means of forum communication for young sexual minoritized men can support HIV testing behaviors and improve social connectedness, which may be an issue for youths who do not have affirming social support. A text messaging–based program designed for young sexual minoritized men aged 14 to 18 years incorporated an embedded text buddy feature. This feature aimed to pair 2 intervention participants, allowing them to exchange text messages throughout the program. The program received positive feedback from participants, indicating high acceptance and engagement [46]. Interestingly, this component was used more intensively by sexually inexperienced compared to sexually experienced youths, suggesting that young sexual minoritized men who are sexually inexperienced may have smaller social networks than experienced youths [47]. However, messages between intervention participants or social forums are challenging to implement given the oversight required to monitor for inappropriate content [48]. It is possible that currently available mHealth apps with similar chat features have attributes that are less desirable for young sexual minoritized men. It is interesting to note many youths agreed that a deidentified forum such as the one used in our study increased their engagement and participation, as many participants were appreciative of one another and resonated with personal stories that were shared. Future mHealth technologies might therefore consider a social forum or a similar digital method in which youths are prompted and conversations are facilitated. It has been well documented that digital features give users a sense of ownership and promote their participation in the mHealth intervention [50].

Through our analysis, we also identified several design components of a mobile app for meeting the social and health needs of young sexual minoritized men. First, participants noted that mHealth apps should be mindful of how much medical jargon is presented, as medical literacy may limit a participant’s ability to benefit from this expanded access. Although participants agreed HIV should not be the sole focus of the app, they were strong advocates of being able to access information about HIVST, PrEP, and sexually transmitted infection information.

Finally, given the recent and sudden transition to digital care prompted by the COVID-19 pandemic, mHealth apps should consider how web-based communication channels have changed and how users engage with them. It is unsurprising that the COVID-19 pandemic has been accompanied by a deluge of complex and changing information, with many studies now reporting the negative effects of constantly browsing, forwarding, and sharing information and experiences related to COVID-19, such as social media fatigue [48]. Many participants liked the idea of consolidating essential COVID-19 related information with HIV testing, such as whether in-person appointments or vaccinations were required for select services. Thus, findings from our study provide timely insights into potential strategies to enhance engagement in mobile technologies, as well as offering ways to integrate clinical and behavioral components.

### Limitations

Caution should be exercised when interpreting our results. First, we recruited a convenience sample on the internet, which might have introduced selection bias, potentially limiting the generalizability of our study findings. Additionally, inherent limitations of the focus group methodology, such as group size and maintaining focused discussions, should be acknowledged. Our study’s generalizability is limited to youths with internet access, which may not represent those with restricted technology access. However, we believe that certain key findings (eg, accessibility and potential for web-based dissemination) can inform the development and dissemination of web-based technologies. This could include printed social marketing campaigns and modified in-person events. Our study, however, had limitations regarding the underrepresentation of gender minorities among participants. Therefore, further research is crucial to gain a comprehensive understanding of the design characteristics for mHealth technologies within this population. Additionally, the focus group discussions were conducted using a chat-based format, which may have limited the facilitators’ ability to effectively probe by using nonverbal cues. It is important to acknowledge that qualitative findings inherently involve subjectivity [42-44]. However, to ensure credibility and dependability of this study’s findings, various researchers were involved throughout the project, from focus group discussion facilitation to coding. We strengthened the credibility of our findings by conducting rigorous thematic content analysis and interpreting the data through extensive discussion and validation by the research team [44].

### Conclusions

This study extends the literature by eliciting young sexual minoritized men’s perspectives of mHealth apps and suggestions on web-based content and design. Findings from this work can be used to inform the development of a convenient, accessible, and easily disseminated tool to help prevent HIV infection and support the implementation of PrEP among young sexual minoritized men.

## Abbreviations

CAS: condomless anal sex
HIVST: HIV self-testing
mHealth: mobile health
PrEP: pre-exposure prophylaxis

## Data Availability

Data are not accessible to the public to minimize the risk of loss of confidentiality. Data are available upon request to the Administrative Core of the Health Intervention Sciences Group or Center for AIDS Intervention Research. Individuals who meet criteria for access to deidentified data should contact the principal investigator (SAJ) or Kevin Brown to facilitate data transfer.
